# Variables associated with job satisfaction among mental health professionals

**DOI:** 10.1371/journal.pone.0205963

**Published:** 2018-10-18

**Authors:** Marie-Josée Fleury, Guy Grenier, Jean-Marie Bamvita, Lambert Farand

**Affiliations:** 1 Department of Psychiatry, McGill University, Montreal, Quebec, Canada; 2 Douglas Mental Health University Institute Research Centre, Montreal, Quebec Canada; 3 Department of Health Administration, Policy and Evaluation School of Public Health, University of Montreal, Montreal, Quebec, Canada; Binghamton University, UNITED STATES

## Abstract

Recent mental health (MH) reforms have had a sharp impact on practices among MH professionals. A deeper understanding of factors contributing to their job satisfaction, in this context, may help improve quality and continuity of care. The purpose of this study was to identify variables associated with job satisfaction for 315 MH professionals in Quebec (Canada) after implementation of wide-ranging MH reforms. Job satisfaction was measured with the Job Satisfaction Survey. Independent variables were conceptualized within five domains: 1) Professional Characteristics, 2) Team Attributes, 3) Team Processes, 4) Team Emergent States, and 5) Organizational Culture. Univariate, bivariate and multivariate analyses were performed. Job satisfaction was significantly associated with absence of team conflict, stronger team support, better team collaboration, greater member involvement in the decision-making process (Team Processes), Affective commitment toward the team (Team Emergent States), as well as lack of a market/rational culture (Organizational Culture). Job satisfaction was strongly related to team processes and, to a lesser extent, team emergent states.

## Introduction

The renewal of mental health (MH) systems, with a goal of improving quality and continuity of care, has been on the agenda in several countries since the 2000s. The province of Quebec (Canada) exemplifies this international trend: the 2005 Quebec MH reform aimed to strengthen primary care MH services, and improve collaboration among psychiatrists and general practitioners through shared care initiatives in order to improve the integration of primary care and specialized MH services [1]. These changes had considerable impact on the practices of MH professionals. Those transferred from specialized services to primary care settings faced increased resource scarcity, for example. Asking professionals with different values, experiences, and practices to work together may have led to friction. While client satisfaction was the central focus of MH reform [2], job satisfaction among MH professionals was, and remains, a major consideration.

Job satisfaction, defined as “a pleasurable or positive emotional state resulting from the appraisal of one’s job or job experiences” [3], constitutes a key outcome of successful teamwork in the health field [4, 5]. Professionals who experience greater work satisfaction are better motivated to work together as a team, and collaborate with other groups or organizations. Job satisfaction also correlates with better quality of care [6]. A happy worker is more likely to resolve problems and make better decisions [7], which may, in turn, minimize or prevent errors [8]. Moreover, health care professionals whose services have a positive impact on clients experience greater satisfaction [9]. Job satisfaction also tends to reduce absenteeism [10], staff turnover [4], and burnout [11], deterring substance use and mental or physical distress [11] while contributing to staff dependability. By contrast, too many unsatisfied professionals may pose a serious barrier to successful healthcare reform [12, 13]. Dissatisfied professionals express more negative feelings toward their clients [14], provide less adequate service, and tend to terminate employment prematurely, with adverse effects for both service continuity and client MH recovery.

Job satisfaction is related to several categories of variables associated with the individual characteristics of professionals, expectations regarding workplaces and organizations, as well as relationships with other professionals [15, 16]. The prevailing organizational culture, described as the foundational assumptions, values and beliefs that underpin an organization [17] and are upheld by each of its members [18], also influences job satisfaction among professionals [5]. Several conceptual models have classified variables related to job satisfaction. The motivation-hygiene theory [19], for instance, distinguishes between variables related to job satisfaction, and to job dissatisfaction. While factors intrinsically related to the job (e.g. recognition, opportunities for personal achievement, work challenges) influence job satisfaction, other, more extrinsic, factors (e.g. interpersonal relationships, salaries) may be associated with job dissatisfaction [20]. According to the Input-Mediator-Outcomes-Input (IMOI) Model, individual characteristics (e.g. age, profession) are nested within team attributes (e.g. type of team, size) and, in turn, within the organizational context (e.g. organizational culture). These variables, considered as inputs, influence two kinds of mediators: team processes, or the methods adopted by team members to work together and accomplish tasks (e.g. ways of dealing with conflict on teams) and team emergent states, or the outputs of teamwork, including motivation, cognition, and emotions such as trust [21]. Team processes and emergent states both generate job satisfaction [22], among other outcomes. Similarly, the heuristic Integrated Team Effectiveness Model (ITEM) states that task design variables (e.g. type and composition of team) are influenced by external environments including organizational context, culture, and the social or policy context, which teams manipulate, in turn [23]; potentially impacting team effectiveness, or the ability to reach objectives [24]. According to studies of job satisfaction based on the IMOI [22] and ITEM [23] models, associated variables may be grouped within five domains: 1) Professional Characteristics, 2) Team Attributes, 3) Team Processes, 4) Team Emergent States, and 5) Organizational Culture.

Professional Characteristics: Job satisfaction has been studied in terms of individual variables such as age, gender, type of profession, and seniority. For example, job satisfaction among physicians was found to be usually high [25] as opposed to nurses [26], social workers [25, 27], and other health care professionals [4]. High satisfaction among physicians may be due to perceptions that their tasks are relatively complex and of great importance [28]. As well, physicians enjoy high status in the professional hierarchy [29]. By contrast, younger professionals and those with less professional seniority have generally reported lower job satisfaction [12]. Significant associations between job satisfaction and gender have not yet been identified.

Team Attributes: These included group composition that may influence team effectiveness [30] and job satisfaction, more indirectly, due to the presence of professionals with different values and practices. Staff shortages on teams may also hinder job satisfaction [26], due to increased tasks and heavier caseloads. A comparative study of job satisfaction among psychiatric nurses [16] found the greatest satisfaction among those working in forensic services, which suggests that the type of clientele may influence professional job satisfaction. Moreover, job satisfaction was higher in smaller units where patients had less severe illnesses [31]. MH professionals dealing with aggressive patients, or those with severe MH and substance use disorders experienced high levels of stress [25].

Team Processes: Studies have investigated team processes extensively in relation to job satisfaction [25]. According to a literature review on job satisfaction, stress and burnout, positive collaboration among professionals, and frequent contact, were predominant factors in job satisfaction among community MH workers [4, 25, 32]. Job satisfaction also correlated with team and organizational support [26, 33], participation in decision-making [16, 26], team autonomy [25, 28], absence of conflict [16], and self-efficacy, i.e. “one’s belief [in his/her] ability and capacity to accomplish a task or cope with environmental demands” [34]. Furthermore, as knowledge related strongly to competent teamwork [35], there is a likely association between knowledge production and job satisfaction.

Team Emergent States: Variables associated with job satisfactions among team emergent states included team climate [16, 26, 36], trust, and affective commitment toward the team. [16, 36] Yet the association between job satisfaction and belief in the advantages of multidisciplinary collaboration has apparently not been assessed. The importance of this variable for interdisciplinary collaboration [37], and for job satisfaction, justifies further investigation.

Organizational Culture: Organizational culture is distinct from team climate, which refers to attitudes, norms and expectation in a specific context, and is also associated with job satisfaction [38]. Job satisfaction varies according to the prevailing corporate culture [5, 39], which is often defined along two axes: flexibility/stability in approaches to work, and internal/external focus of the governing structure [40]. The dominant organizational cultures fall into the following categories: a) family/clan culture (flexibility-internal focus), b) entepreunarial culture (flexibility-external focus), c) market/rational culture (stability-external focus), or d) hierarchical culture (stability-internal focus) [18]. Each culture highlights particular values: loyalty, development, participation and staff empowerment in the family/clan culture; listening capacity with clients, innovation and risk-taking in the entrepreneurial culture; competition, results-orientation, and achievement of measurable goals in the market/rational culture; and coordination, formalization, stability and efficiency in the hierarchical culture [18, 41]. Job satisfaction is expected to be higher in a family/clan culture, moderate in an entrepreneurial or market/rational culture, and lower in a hierarchical culture [42].

Overall job satisfaction in the health field has been the subject of various studies, mainly focused on specific groups of professionals such as nurses [15, 20, 43–45], social workers [27] or physicians [11]. Other research has assessed job satisfaction among health workers within specific settings such as community MH teams [25, 46] and acute care hospitals [4], or at particular stages of professional life (e.g. early career) [14]. No known studies have assessed job satisfaction among various types of MH professionals working in different settings (e.g. primary care, specialized MH services), however, or across different local health service networks. The new primary care MH teams developed in the context of the Quebec reforms often included professionals transferred from specialized MH services. where they had enjoyed considerable organizational and material support [33]. The shift of these MH professionals to primary care may have created some degree of job dissatisfaction, which warrants investigation.

A deeper understanding of the determinants of job satisfaction among MH professionals in the Quebec context, might contribute to better quality and continuity of care for service users. As such, this study aimed to identify variables linked to job satisfaction among 315 MH professionals in Quebec (Canada) following the implementation of a major MH reform. Based on a conceptual model adapted from the IMOI Model [22] and ITEM [23] as well as the literature reviewed above, we hypothesized that job satisfaction would more likely be associated with 1) Team Processes and 2) Team Emergent States than with Professional Characteristics, Team Attributes or Organizational Culture.

## Materials and methods

### Study design and data collection

This cross-sectional study stems from a comprehensive evaluation of the 2005 Quebec MH reform [1], which set up 95 local health services networks, and merged general hospitals, nursing homes, and local community service centers (all public services) to create a health and social service center (HSSC) within each network. The HSSCs handle multiple functions including the supervision of care quality within primary MH care services, as well as the coordination of primary care and specialized MH professionals within the respective networks.

The sample consisted of MH professionals selected from four local health service networks. The networks were chosen to include diverse geographical areas (urban or semi-urban), demographic characteristics (populations ranging from 135,000 to 300,000), and diversity of services offered. Two networks were located in a large metropolitan area inhabited by half the Quebec population, with specialized services available from a MH university institute. The third network was located in the provincial capital region, where another MH university Institute is located. The fourth network was located in a remote, semi-urban area, where a general hospital provided MH services. All the networks offered primary MH care, as well.

An advisory committee, including representatives from the four networks, provided the researchers with a list of MH team managers for each network, who, in turn, furnished lists of all MH professionals from their respective teams. To be eligible for the study, professionals had to be members of a MH team that included three or more professionals representing at least two disciplines, based on previous research related to teamwork, and taking into account a minimum of interaction patterns required by the complexity of MH care [47, 48]. MH professionals were approached by the research team via email, or telephone, regarding their potential interest and involvement in the research.

A self-administered questionnaire was mailed to 466 eligible MH professionals. Questionnaire items included 13 standardized scales on multidimensional aspects of teamwork, and nine questions on individual characteristics (e.g. age, gender). Data collection occurred between May and November 2013, following three recruitment drives in the four networks. Team managers who employed professionals recruited to the study (n = 49, October 2013 and June 2014), were themselves recruited by email or telephone during the same period. Managers received a second questionnaire, which included information available in administrative databases, regarding eight elements: 1) manager characteristics (e.g. age, gender); 2) client profiles (e.g. proportion of heavy service users); 3) team attributes (e.g. size, setting); 4) clinical activities (e.g. clinical approaches); 5) organizational culture; 6) network integration strategies (e.g. service agreements); 7) frequency and satisfaction of interactions with other network teams and organizations; and 8) MH services in the network. Only data on client profiles, team attributes and organizational culture were relevant to this study. Both manager and MH professional questionnaires were pre-tested by eight professionals, two from each network. All study participants signed a consent form. The Douglas Mental Health University Institute research ethics committee approved the multi-site research protocol (MP-IUMD-11037).

### Conceptual framework, variables and standardized scales

**Fig 1:**
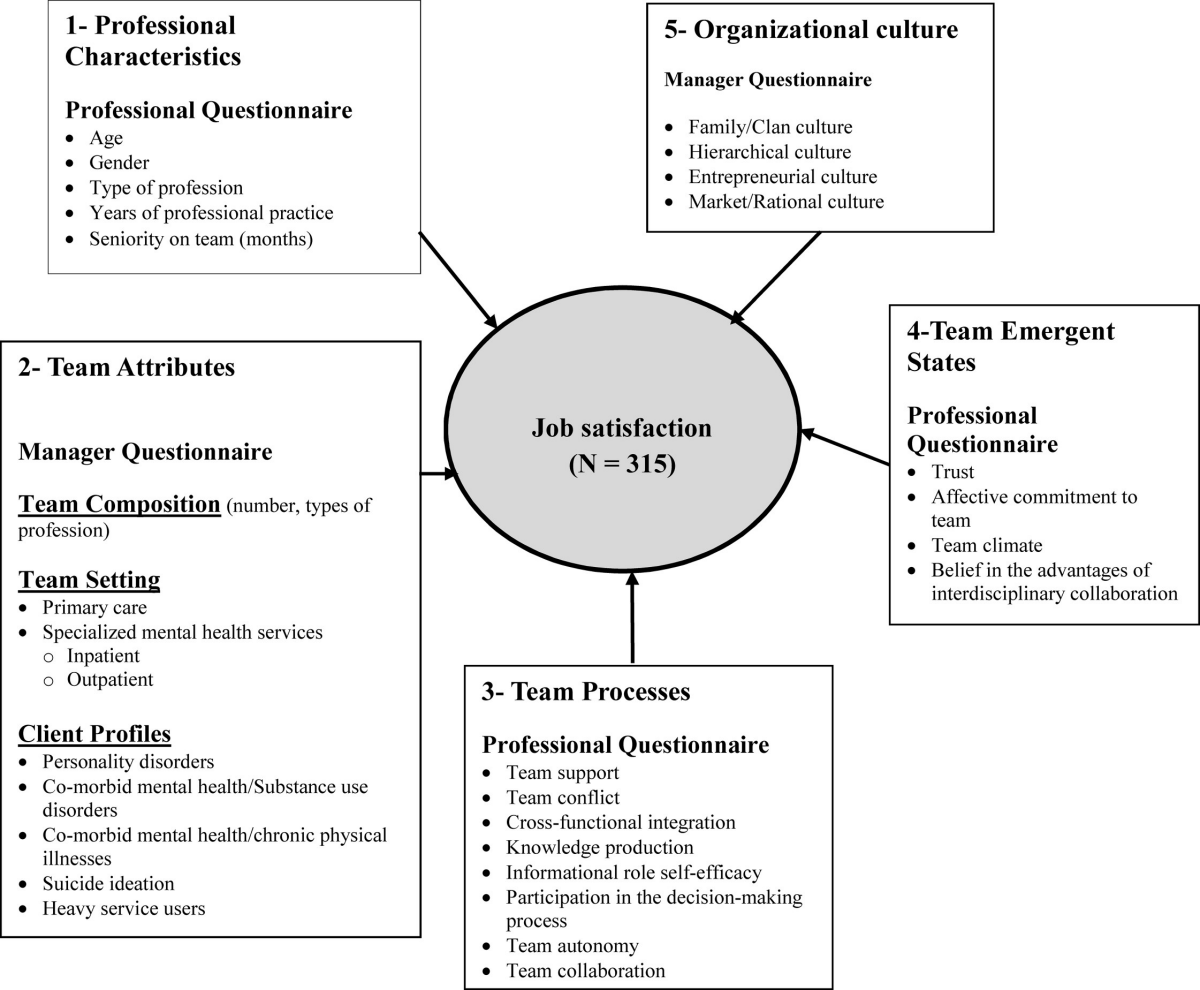
Conceptual framework. Independent Variables were grouped into five domains: 1) Professional Characteristics; 2) Team Attributes; 3); Team Processes; 4) Team Emergent States; and 5) Organizational Culture.

The conceptual framework used in this study was adapted from the IMOI Model [22] and the ITEM [23], with independent variables organized within the five following domains: 1) Provider Characteristics, 2) Team Attributes, 3) Team Processes, 4) Team Emergent States, and 5) Organizational Culture (**Fig 1**). Data regarding Professional Characteristics, Team Processes, and Team Emergent States (Fig 1) were mainly drawn from the MH professional questionnaire, while data on Team Attributes and Organizational Culture came from the manager questionnaire.

The dependent variable “Job satisfaction” was assessed using the French-language version of the Job Satisfaction Survey [49]. The original instrument consisted of 36 items grouped into nine sub-dimensions with Likert scale responses (Cronbach alpha = 0.91 for the global scale, between 60–78 according the sub-dimension [49]). We eliminated 16 items and 4 sub-dimensions that dealt with remuneration, as this issue did not apply to the Quebec public healthcare system. Measures of Cronbach’s alpha for the scale used in this study ranged from 0.63 (for relations with co-workers) to 0.78 (for job conditions).

Independent variables on Professional Characteristics were: Age, Gender, Type of profession, Years of professional practice, and Seniority on the team. Types of profession included four groups: 1) Medical (psychiatrist, pharmacist, general practitioner), 2) Nurse, 3) Psychosocial (social worker, psychologist), and 4) General (technician, clerk).

Independent variables for Team Attributes were: Team composition (number, types of professionals), Team setting (Specialized outpatient MH teams, Specialized inpatient MH teams, Primary care teams) and Client profile (proportions with: severe MH disorders, personality disorders, co-morbid MH and substance use disorders, co-morbid MH disorders and chronic physical illnesses, suicide ideation, and heavy service users).

Independent variables for Team Processes were measured using eight standardized scales that assessed: Team support, Team conflict, Cross-functional integration (e.g. integration among various disciplines), Knowledge production, Informational role self-efficacy (i.e. belief in the ability to complete a task or deal with exterior demands), Participation in the decision-making process, Team autonomy, and Team collaboration. These scales were translated into French, and validated, with the exception of the scales for Informational role self-efficacy and Team collaboration that were originally developed in French.

Independent variables for Team Emergent States were measured using four standardized scales that assessed Trust, Affective commitment toward the team, Team climate, and Belief in the advantages of interdisciplinary collaboration. The relevant scales were translated into French, and validated, except for the scale on Belief in the advantages of interdisciplinary collaboration, which originally appeared in French.

Finally, Organizational Culture, as measured by team managers, was measured using the Organizational Culture Assessment Instrument [41], which consists of six questions. Reponses include four possible choices, with each response distributed among a possible 100 points. Organizational Cultures were classified as 1) Family/clan culture; 2) Entrepreneurial culture; 3) Market/rational culture; and 4) Hierarchical culture. **Table 1** presents details of the standardized scales used in the study and their Cronbach’s alpha scores at original validation, and in the present study.

**Table 1:** Description of standardized instruments included in the study.

Measures	References	Description	Cronbach’s Alpha Coefficients from the Original Validation	Cronbach’s Alpha Coefficients in the Present Study
**Dependent Variable **				
**Job Satisfaction**	[49]	20 items; 5 sub-dimensions	0.91 (global scale) 0.60–0.78 (sub-dimensions)	0.63–0.78
**Independent Variables**				
*For Team Processes*:				
Team support	[50]	4 items	0.72	0.84
Team conflict	[51]	9 items	0.93–0.94	0.84–0.91
Cross-functional integration	[52]	9 items	N.A.	0.95
Knowledge production	[53]	5 items	0.71–0.95	0.95
Informational role self-efficacy	[54]	5 items	0.93	0.93
Participation in the decision-making process	[55]	3 items	0.88	0.90
Team autonomy	[55]	3 items	0.76	0.81
Team collaboration	[56]	14 items	0.77–0.912	0.83–0.94
*For Team Emergent States*:				
Trust	[57]	4 items	89	0.92
Affective commitment toward the team	[58]	5 items	0.86–0.92	0.91
Team climate	[59]	19 items; 4 sub-dimensions	0.60–0.84	0.84–0.93
Belief in the advantages of interdisciplinary collaboration	[37]	5 items	0.92	0.92
*For Organizational Culture*:				
Organizational culture assessment instrument	[41]	6 items	N.A.	N.A.

### Analyses

Statistical analyses were run with SPSS, 24th edition. Missing values accounted for less than 5% per variable, and were randomly distributed. They were treated using Expectation Maximization, a multiple imputation technique. Following preparation of the database, we conducted univariate, bivariate and multivariate analyses. Univariate analyses included central tendency measures (mean value) for continuous variables and frequency distributions (numbers and percentages) for categorical variables. Normality assumptions were assessed for the dependent variable. Bivariate analyses were then performed using simple linear regression, and ANOVA t-tests, with the alpha value set at 0.10. Variables significantly associated with the dependent variable were used to build the multiple linear regression model, with alpha set at 0.05. The variance explained (R^2^) by the model, and goodness of fit (F test and p value), were also calculated.

To assess the effects of MH networks and MH teams on Job satisfaction, we ran a multilevel analysis using independent variables significantly associated with the dependent variable (Job Satisfaction) in the multiple linear regression model. An unconditional model was first run, using only the dependent variable. Intra-class correlation was then calculated, estimating the proportion of total variance in Job satisfaction attributable to the networks and to teams. Fixed and random coefficients were then tested, step by step, using maximum likelihood estimation. The fit was assessed for each model using maximum likelihood (-2LL), Akaike's information criterion (AIC) and Schwarz's Bayesian criterion (BIC). The different models were compared one to another, with a smaller value indicating improvement in model fit. Covariance parameters were estimated using the Wald Z test, which assesses whether variability in the intercepts was significantly different among MH teams and the networks. As the Wald Z test is considered unreliable [60], results were not viewed as conclusive even when testing produced a non-significant P value. Finally, a multilevel model was generated and compared to the multiple linear regression model in order to highlight differences and decide whether the networks or teams contributed to a better assessment of Job satisfaction among MH professionals.

## Results

Of 466 MH professionals recruited to the study, 315 participated, for a 68% response rate. The 315 MH professionals came from 49 MH teams, with six members on average (range from three to 16). In all, the teams represented nine different health care groups. Specialized service groups included hospital units, day hospitals, assertive community treatment programs, outpatient clinics, and rehabilitation programs. Primary care groups included evaluation units, local community service centers, basic teams and intensive case management programs. An average of 35 professionals (ranging from 30 to 55) worked in each group. Response rates for the networks were 64% (157/247) for networks 1 and 2; 59% (117/198) for network 3; and 80% (44/55) for network 4.

There were no differences between participant and non-participant MH professionals on the distributions for team type (χ^2^ [1, N = 466] = 0.79; *p* = 0.68), or gender (χ^2^ [1, N = 466] = 0.03; *p* = 0.87). The mean age was 43, and mean seniority on clinical teams was three years. Women outnumbered men by more than two to one (70% versus 30%). Most participants (78%) worked full-time, while 22% worked part-time. More than half (55%) were Psychosocial professionals, followed by Nurses (30%), General workers (11%) and Physicians (4%). Most worked in Specialized outpatient MH teams (56%), with the remainder in either Primary care teams (32%) or Specialized inpatient MH teams (12%). Almost three-quarters of the teams (74%) operated in the three urban settings, versus 16% in the semi-urban setting. The outcome variable “Job satisfaction” had a mean score of 24.8 (range: 11.3–35.0; SD: 3.6) and was normally distributed (skewness:–.037; kurtosis: .332).

Regarding team managers, 41 participated out of 49 invited to the study, for a response rate of 84%. Response rates within the networks were 75% for networks 1 and 2 (18/24); 94% for network 3 (16/17); and 88% for network 4 (7/8). Comparative analyses revealed no differences between respondent and non-respondent managers on gender (Pearson chi-square = .966; df = 1; Fisher’s exact test two-sided *p* = .663); or type of health care setting (Pearson chi-square = 1.861; df = 1; Fisher’s exact test two-sided *p* = .245.) Of participating managers, 71% were female, 62% were members of Specialized MH teams, and 38% worked in Primary care teams. The mean age was 44, and mean seniority in the team four years.

**Table 2** presents 15 variables linked to job satisfaction in the bivariate analysis. Four of these were positively related to Team Processes: Team support, Cross-functional integration, Knowledge production, Informational role self-efficacy; whereas one variable, Team conflict, was negatively related. Four variables in Table 2 were related to Team Emergent States: Trust, Affective commitment toward the team, Team climate, and Belief in advantages of interdisciplinary collaboration. Three variables emerged under Team Attributes: Proportion of personality disorder in the clientele, Specialized inpatient MH teams and, marginally, Proportion of co-morbid MH disorder and chronic physical illnesses in the clientele. Job satisfaction was associated with two types of Organizational Culture, as measured by team managers: Market/rational culture and, negatively, with Family/clan culture. Finally, only one variable among Professional Characteristics (Male gender) was related to Job satisfaction.

**Table 2:** Participant characteristics and unadjusted associations with job satisfaction (N = 315.

	Model		Frequency Distribution				Bivariate Analyses	
			Min	Max	n/Mean	%/SD	Standardized Coefficients Beta	P
1. Professional Characteristics		Gender (n/%)						
		*Female*			219	69.5		1.00
		*Male*			96	30.5	.125	.027
2. Team Attributes	Types of health care teams (n/%)							
	Primary health care teams				101	32.1		1.00
	Specialized outpatient mental health (MH) teams				176	55.9	.066	.288
	Specialized inpatient MH teams				38	12.1	.126	.043
	Proportion of personality disorder in the clientele (Mean/SD)		2.0	90.0	30.9	21.1	–.133	.018
	Proportion of co-morbid MH disorder and chronic physical illnesses in the clientele (Mean/SD)		2.0	93.0	33.5	22.5	.115	.066
3. Team Processes	Team support (Mean/SD)		1.0	7.0	4.8	1.2	.486	< .001
	Team conflict (Mean/SD)		3.0	21.0	9.0	2.9	–.380	< .001
	Cross-functional integration (Mean/SD)		1.1	7.0	4.3	1.1	.443	< .001
	Knowledge production (Mean/SD)		1.0	7.0	4.0	1.2	.311	< .001
	Informational role self-efficacy (Mean/SD)		16.0	100.0	81.1	14.4	.160	.004
	Participation in the decision-making process (Mean/SD)		1.0	7.0	5.0	1.4	.461	< .001
	Team autonomy (Mean/SD)		1.0	7.0	4.9	1.3	.233	< .001
	Team collaboration (Mean/SD)		8.5	28.0	19.3	3.8	.492	< .001
4. Team Emergent States	Trust (Mean/SD)		1.0	7.0	5.2	1.2	.392	< .001
	Affective commitment toward the team (Mean/SD)		1.0	7.0	4.9	1.2	.415	< .001
	Team climate (Mean/SD)		7.9	27.8	20.5	3.4	0.529	< .001
	Belief in advantages of interdisciplinary collaboration (Mean/SD)		3.0	7.0	6.2	0.7	.276	< .001
5. Organizational Culture	Organizational culture (Mean/SD)							
	Family/clan culture		60.0	355.0	209.9	66.6	–.191	.001
	Market/rational culture		25.0	200.0	110.0	43.4	.130	.038

**Table 3** presents the multiple linear regression model for the study. Four variables were identified in the model as independently and positively associated with Job satisfaction: Team support, Team collaboration, Participation in the decision-making process (Team Processes), and Affective commitment toward the team (Team Emergent States). Two variables were negatively associated with Job satisfaction: Team conflict (Team Processes) and Market/rational culture (Organizational Culture). No variables under Professional Characteristics or Team Attributes were linked to Job satisfaction in the final model. This model explained 45% of the total variance and had acceptable goodness of fit.

**Table 3:** Variables independently associated with job satisfaction: Multiple linear regression.

Model	Unstandardized Coefficients		Standardized Coefficients	t	P	95.0% Confidence Interval for B		Collinearity Statistics	
	B	Std. Error	Beta			Lower Bound	Upper Bound	Tolerance	VIF
Intercept	17.659	1.269		13.920	<0.0001	15.163	20.156		
**Team Processes**									
Team conflict	-0.307	0.054	-0.249	-5.683	<0.0001	-0.413	-0.201	0.922	1.085
Team support	0.846	0.149	0.275	5.693	<0.0001	0.554	1.139	0.757	1.321
Team collaboration	0.142	0.050	0.151	2.807	0.005	0.042	0.241	0.609	1.643
Participation in the decision-making process	0.521	0.136	0.197	3.844	<0.0001	0.254	0.788	0.671	1.489
**Team Emergent States**									
Affective commitment toward the team	0.316	0.151	0.108	2.094	0.037	0.019	0.613	0.662	1.510
**Organizational Culture**									
Market/rational	-0.005	0.002	-0.098	-2.272	0.024	-0.010	-0.001	0.940	1.064
Total variance explained: R^2^	44.6								

**Table 4** presents the multilevel model. A tentative model assessing the effect of MH networks did not converge and was eliminated. The multilevel model assessed the contribution of MH teams to MH professional job satisfaction. The unconditional model (Model 1) yielded an ICC of 10.2%. The one variable pertaining to the team level, Market/rational culture (as measured by team managers), was significantly and negatively associated with Job satisfaction. Among variables at the individual level, Team conflict was negatively associated with Job satisfaction, whereas four other variables were positively associated: Team support, Team collaboration, Participation in the decision-making process and Affective commitment toward the team. A tentative model with random slopes did not converge and was eliminated. The Wald Z test of the final model was not significant (P = 0.548). Comparison with the multiple linear regression model shows that the two models are quite similar.

**Table 4:** Variables significantly associated with job satisfaction among mental health professionals: Multilevel analysis.

		Model 1: Unconditional			Model 2: Contextual variables: Mental health managers variables			Model 2: Overall model: Mental health professional and manager variables						
*Estimates of Fixed Effects*	Parameter	Estimate	t	P	Estimate	t	P	Estimate	Std. Error	df	t	P	95% Confidence Interval	
													Lower Bound	Upper Bound
	Intercept	24.792	95.012	<0.0001	26.982	34.114	<0.0001	17.705	1.283	166.745	13.802	<0.0001	15.172	20.238
	Market/rational culture				-0.011	-2.911	0.006	-0.005	0.002	39.013	-2.211	0.033	-0.010	<0.0001
	Team conflict							-0.309	0.054	244.833	-5.717	<0.0001	-0.416	-0.203
	Team support							0.839	0.148	282.887	5.661	<0.0001	0.547	1.131
	Team collaboration							0.143	0.050	314.318	2.856	0.005	0.044	0.241
	Participation in the decision-making process							0.522	0.135	290.126	3.868	<0.0001	0.256	0.788
	Affective commitment toward the team							0.320	0.150	301.753	2.131	0.034	0.025	0.615
*Random Effects*: *Estimates of Covariance Parameters*		Estimate	Wald Z	P	Estimate	Wald Z	P		Estimate	Std. Error	Wald Z	P	95% Confidence Interval	
													Lower Bound	Upper Bound
	Residual	11.718	11.453	<0.0001	11.706	11.465	<0.0001		6.912	0.594	11.641	<0.0001	5.841	8.179
	Intercept [subject = Mental Health Teams]	1.331	1.834	0.067	0.855	1.391	0.164		0.138	0.252	0.548	0.584	0.004	4.938
Intraclass correlation (ICC)		0.102			0.068				0.020					
*Information Criteria*	-2 Log Likelihood	1695.060			1687.169				1508.737					
	Akaike's Information Criterion (AIC)	1701.060			1695.169				1526.737					
	Hurvich and Tsai's Criterion (AICC)	1701.137			1695.299				1527.327					
	Bozdogan's Criterion (CAIC)	1715.317			1714.180				1569.510					
	Schwarz's Bayesian Criterion (BIC)	1712.317			1710.180				1560.510					

## Discussion

The findings confirm our hypothesis that Job satisfaction among MH professionals is more closely related to Team Processes, as represented by four out of six variables in the final model. In addition, the four strongest determinants of Job satisfaction were all Team Processes variables: less Team conflict, more Team support, more Team collaboration and more Participation in the decision-making process.

The main determinant of job satisfaction was less Team conflict, which coincides with previous studies and confirms the Herzberg theory, which identified job dissatisfaction as strongly related to poor relationships among co-workers [19]. Relational conflicts exert the greatest influence on job satisfaction [61]. Other literature further suggests that team conflict is a significant predictor of burnout among MH professionals. [4, 5, 16, 62] By contrast, having positive contact with co-workers was identified as one of the most important rewards offered by teamwork among community MH workers [25]. When relationships among team members are positive, task-related conflict may be beneficial as long as it enhances problem-solving [61, 63]. Discussion on points of disagreement may lead to better decisions [64], as such communication tends to promote greater understanding of issues among staff and a desire to reach solutions that reflect group consensus [61].

Lack of team support is also related to job dissatisfaction as a significant source of workplace stress [36]. Adequate material and human resources may support teams by reducing the workload of individual members; while rewards stimulate individual development [6]. The available literature suggests that professionals who receive adequate social and supervisory support in the workplace are more satisfied with their jobs [26], and less likely to resign prematurely [65].

Close collaboration is another characteristic of high-functioning teams, and promotes individual job satisfaction [23]. Collaboration promotes trust among team members [63]. Teams that develop more collaborative communication practices are less likely to experience conflict regarding work-related tasks [61].

Involving every team member in decision-making promotes open debate and problem-solving [66], while fostering professional engagement and a sense of individual responsibility [67], as well as job satisfaction [26]. By contrast, professionals with little decision-making latitude are more likely to be affected by stress, burnout, and mental distress [25, 27].

While Team Emergent States variables related more to Job satisfaction than did Professional Characteristics, Team Attributes or Organizational Culture, Affective commitment toward the team was the only variable under Team Emergent States to emerge in the final models. The association between Affective commitment toward the team and Job satisfaction is, however, interesting, considering that the primary care teams were relatively new, and often assembled by transferring numerous professionals from specialized services. Affective commitment toward the team facilitates staff retention, while reinforcing the personal identification of professionals to their institutions, another essential condition for Job satisfaction [68, 69].

One innovative finding from our study was identification of a negative association between Job satisfaction and the Market/rational culture, as measured by team managers, at the organizational level. Strongly aligned with environmental demands [18], the market/rational culture is better suited than other cultures for meeting economic and strategic challenges [18], not unlike those precipitated by healthcare reforms such as that which occurred in Quebec. However, research indicates that professionals working in organizations characterized by market/rational cultures often view themselves as relatively ineffective, and experience lower rates of job satisfaction [39]. From this perspective, the market/rational culture may also increase stress and professional rivalry. The highest job satisfaction scores have generally been associated with the family/clan culture, with its focus on team collaboration and shared decision-making [39].

Finally, it should be recalled that the bivariate analysis revealed a link between Male gender (Professional Characteristics) and certain Team Attributes (Specialized impatient MH teams, Proportion of personality disorder in the clientele); although none of these associations emerged in the final models. We may conclude that the strength of Team Processes variables in explaining Job satisfaction among MH professionals tended to overshadow, and eliminate, the variables under Professional Characteristics and Team Attributes.

This study has some notable limitations. First, the data are cross-sectional and, as such, could not be used to infer cause and effect relationships with Job satisfaction. For example, it was impossible to determine whether Job satisfaction promoted Belief in the advantages of interdisciplinary collaboration, or vice versa. Second, some variables previously identified as associated with Job satisfaction, such as salary and workload [4], were not available for this study. Third, the variables in this study were all based on self-report measures, which may have produced slightly different results than if objective measures were used. Finally, the results may not be generalized to other Quebec health networks or to jurisdictions where health services, or the healthcare system itself, are organized differently than those in the present study. Further research is needed to replicate these findings.

## Conclusion

This study tested a comprehensive set of variables related to Professional Characteristics, Team Attributes, Team Processes, Team Emergent States, and Organizational Culture with the aim of establishing links with Job satisfaction among MH professionals. The findings confirm that Job satisfaction among MH professionals is strongly related to Team Processes (e.g. Team conflict, Team support), and, to a lesser degree, to Team Emergent States in terms of a single variable: Affective commitment toward the team. Findings further suggest that MH managers should focus on assessing and improving team support, inter-professional collaboration, and the participation of MH team members in decision-making processes. Managers should also seek ways of reducing team conflict in order to increase job satisfaction and, ultimately, ensure better quality and continuity of care. Strong supervisory support contributes to job satisfaction, so may be a critical factor in ensuring stable management in MH teams and greater commitment among professionals toward their teams. Finally, but not least, participation in the decision-making process ensures that the expertise of each MH professional will be fully recognized.

## Supporting information

S1 File Questionaire for mental health professionals. (PDF)

S2 File Summary of the questionnaires for managers. (DOCX)
