# Supplementary material for: Variables associated with job satisfaction among mental health professionals
Source: PLoS One. 2018 Oct 18;13(10):e0205963. doi: 10.1371/journal.pone.0205963 (PMC6193708; doi:10.1371/journal.pone.0205963)
Supplement: S1 File — (PDF) [file pone.0205963.s001.pdf]

37518

1. Informations générales à votre sujet

CODE DU RÉPONDANT

DATE DE PASSATION DU QUESTIONNAIRE

jour mois année

NOM DE VOTRE ÉQUIPE

1) Quelle est votre profession ?

- ○ Médecin généraliste
- ○ Médecin spécialiste
- ○ Infirmière / Infirmier
- ○ Pharmacienne / Pharmacien
- ○ Psychologue
- ○ Technicien / Technicienne
- ○ Travailleur social / Travailleuse sociale
- ○ Ergothérapeute
- ○ Nutritionniste
- ○ Orthophoniste
- ○ Autre (veuillez préciser)

2) Depuis combien d'années / mois exercez-vous cette profession ? Années Mois

3) Depuis combien d'années / mois occupez-vous votre poste actuel dans cet établissement ? Années Mois

4) Depuis combien d'années / mois travaillez-vous dans l'équipe dont il est question dans ce questionnaire? Années Mois

5) Actuellement, est-ce que vous travaillez à temps plein ou à temps partiel dans cette équipe ? (Si à temps partiel, indiquez le nombre d'heures par semaine.)

- ○ Temps plein
- ○ Temps partiel .... heures

6) Quel est votre âge ?

7) Vous êtes :

- ○ Un homme
- ○ Une femme

37518

2. Questions concernant le travail d'équipe et la collaboration interprofessionnelle

a) Familiarité des coéquipiers

	Complètement en désaccord	Très en désaccord	Un peu en désaccord	Ni en désaccord ou en accord	Un peu en accord	Très en accord	Complètement en accord
1. Je suis familier avec les forces et les faiblesses de mes coéquipiers.	○ 1	○ 2	○ 3	○ 4	○ 5	○ 6	○ 7
2. Je suis familier avec les compétences de mes coéquipiers.	○ 1	○ 2	○ 3	○ 4	○ 5	○ 6	○ 7
3. Je suis familier avec la réputation professionnelle de mes coéquipiers.	○ 1	○ 2	○ 3	○ 4	○ 5	○ 6	○ 7
4. Je suis familier avec le rendement au travail de mes coéquipiers.	○ 1	○ 2	○ 3	○ 4	○ 5	○ 6	○ 7
5. Je suis familier avec les valeurs, attitudes et croyances de mes coéquipiers.	○ 1	○ 2	○ 3	○ 4	○ 5	○ 6	○ 7

b) Croyances au sujet des bénéfices de la collaboration interdisciplinaire

	Complètement en désaccord	Très en désaccord	Un peu en désaccord	Ni en désaccord ou en accord	Un peu en accord	Très en accord	Complètement en accord
1. …permet de mieux répondre aux besoins du client ou de l'usager.	○ 1	○ 2	○ 3	○ 4	○ 5	○ 6	○ 7
2. …entraîne une meilleure satisfaction du client ou de l'usager.	○ 1	○ 2	○ 3	○ 4	○ 5	○ 6	○ 7
3. …apporte le soutien que les membres d'une équipe requièrent pour faire leur travail auprès du client ou de l'usager.	○ 1	○ 2	○ 3	○ 4	○ 5	○ 6	○ 7
4. …améliore la qualité de ce qui doit être fait pour le client ou l'usager.	○ 1	○ 2	○ 3	○ 4	○ 5	○ 6	○ 7
5. …favorise la concertation des actions bénéfiques au client ou à l'usager.	○ 1	○ 2	○ 3	○ 4	○ 5	○ 6	○ 7

c) Autonomie de l'équipe

	Complètement en désaccord	Très en désaccord	Un peu en désaccord	Ni en désaccord ou en accord	Un peu en accord	Très en accord	Complètement en accord
1. Les membres de mon équipe sont responsables de déterminer les méthodes, procédures et échéanciers avec lesquels le travail se fait.	○ 1	○ 2	○ 3	○ 4	○ 5	○ 6	○ 7
2. Mon équipe, plus que mon patron, décide de " qui fait quoi " au sein de l'équipe.	○ 1	○ 2	○ 3	○ 4	○ 5	○ 6	○ 7
3. La majeure partie des décisions relatives au travail est prise par les membres de mon équipe plutôt que par mon patron.	○ 1	○ 2	○ 3	○ 4	○ 5	○ 6	○ 7

37518

d) Satisfaction au travail

	Complètement en désaccord	Très en désaccord	Un peu en désaccord	Ni en désaccord ou en accord	Un peu en accord	Très en accord	Complètement en accord
1. Mon patron est compétent dans le travail qu'il effectue.	○ 1	○ 2	○ 3	○ 4	○ 5	○ 6	○ 7
2. Mon patron est injuste avec moi.	○ 1	○ 2	○ 3	○ 4	○ 5	○ 6	○ 7
3. Mon patron montre peu d'intérêt face aux émotions de ses employés.	○ 1	○ 2	○ 3	○ 4	○ 5	○ 6	○ 7
4. J'apprécie mon patron.	○ 1	○ 2	○ 3	○ 4	○ 5	○ 6	○ 7
5. Quand je fais du bon travail, je reçois la reconnaissance que je mérite.	○ 1	○ 2	○ 3	○ 4	○ 5	○ 6	○ 7
6. Je ne sens pas que mon travail est apprécié.	○ 1	○ 2	○ 3	○ 4	○ 5	○ 6	○ 7
7. Il y a peu de récompenses pour les travailleurs de notre organisation.	○ 1	○ 2	○ 3	○ 4	○ 5	○ 6	○ 7
8. Je trouve que mes efforts ne sont pas récompensés comme ils le devraient.	○ 1	○ 2	○ 3	○ 4	○ 5	○ 6	○ 7
9. Plusieurs règles et procédures font qu'il est difficile de faire un bon travail.	○ 1	○ 2	○ 3	○ 4	○ 5	○ 6	○ 7
10. Les efforts que je fais pour améliorer les choses sont souvent bloqués par la bureaucratie.	○ 1	○ 2	○ 3	○ 4	○ 5	○ 6	○ 7
11. J'ai trop de choses à faire dans mon travail.	○ 1	○ 2	○ 3	○ 4	○ 5	○ 6	○ 7
12. J'ai trop de paperasse.	○ 1	○ 2	○ 3	○ 4	○ 5	○ 6	○ 7
13. J'aime les gens avec qui je travaille.	○ 1	○ 2	○ 3	○ 4	○ 5	○ 6	○ 7
14. Je trouve que je dois travailler plus fort à cause de l'incompétence de mes collègues de travail.	○ 1	○ 2	○ 3	○ 4	○ 5	○ 6	○ 7
15. J'apprécie mes confrères de travail.	○ 1	○ 2	○ 3	○ 4	○ 5	○ 6	○ 7
16. Il y a trop de commérages et de conflits au travail.	○ 1	○ 2	○ 3	○ 4	○ 5	○ 6	○ 7
17. Il m'arrive quelquefois de penser que mon travail est sans importance.	○ 1	○ 2	○ 3	○ 4	○ 5	○ 6	○ 7
18. J'aime faire les tâches que je dois accomplir dans mon travail.	○ 1	○ 2	○ 3	○ 4	○ 5	○ 6	○ 7
19. Je suis fier de mon travail.	○ 1	○ 2	○ 3	○ 4	○ 5	○ 6	○ 7
20. Mon travail est plaisant.	○ 1	○ 2	○ 3	○ 4	○ 5	○ 6	○ 7

e) Participation aux décisions

	Complètement en désaccord	Très en désaccord	Un peu en désaccord	Ni en désaccord ou en accord	Un peu en accord	Très en accord	Complètement en accord
1. Comme membre d'équipe j'ai mon mot à dire sur la façon dont l'équipe effectue son travail.	○ 1	○ 2	○ 3	○ 4	○ 5	○ 6	○ 7
2. La plupart des membres de mon équipe ont l'occasion de participer au processus décisionnel.	○ 1	○ 2	○ 3	○ 4	○ 5	○ 6	○ 7
3. Mon équipe est conçue pour que tous puissent participer au processus décisionnel.	○ 1	○ 2	○ 3	○ 4	○ 5	○ 6	○ 7

f) Promotion du rétablissement

	Complètement en désaccord	Très en désaccord	Un peu en désaccord	Ni en désaccord ou en accord	Un peu en accord	Très en accord	Complètement en accord
1. Le programme offre un environnement physique invitant et respectueux (ex. lobby, salles d'attente).	○ 1	○ 2	○ 3	○ 4	○ 5	○ 6	○ 7
2. Le programme offre des services spécifiques ajustés à la culture propre de chaque usager et à ses expériences de vie.	○ 1	○ 2	○ 3	○ 4	○ 5	○ 6	○ 7
3. Le programme encourage les usagers à s'impliquer dans l'évaluation des services et de l'équipe.	○ 1	○ 2	○ 3	○ 4	○ 5	○ 6	○ 7
4. Les usagers sont encouragés à participer au conseil consultatif de l'organisation et aux rencontres de gestion.	○ 1	○ 2	○ 3	○ 4	○ 5	○ 6	○ 7
5. Les usagers sont impliqués dans la formation des employés et dans les programmes de formation de l'établissement.	○ 1	○ 2	○ 3	○ 4	○ 5	○ 6	○ 7
6. Les usagers peuvent changer de clinicien ou de gestionnaire de cas s'ils le veulent.	○ 1	○ 2	○ 3	○ 4	○ 5	○ 6	○ 7
7. Les usagers peuvent facilement accéder à leur dossier médical s'ils le veulent.	○ 1	○ 2	○ 3	○ 4	○ 5	○ 6	○ 7
8. Les usagers sont encouragés à aider les employés au développement de nouveaux groupes, programmes ou services.	○ 1	○ 2	○ 3	○ 4	○ 5	○ 6	○ 7

Les membres de l'équipe ...

	Complètement en désaccord	Très en désaccord	Un peu en désaccord	Ni en désaccord ou en accord	Un peu en accord	Très en accord	Complètement en accord
9. … font un effort concerté pour accueillir les usagers et les aident à se sentir à l'aise dans le programme.	○ 1	○ 2	○ 3	○ 4	○ 5	○ 6	○ 7
10. … encouragent les usagers à avoir de l'espoir et des attentes élevées pour leur rétablissement.	○ 1	○ 2	○ 3	○ 4	○ 5	○ 6	○ 7
11. … n'utilisent pas des menaces, des pots-de-vin ou d'autres formes de pression pour influencer le comportement des usagers.	○ 1	○ 2	○ 3	○ 4	○ 5	○ 6	○ 7
12. … croient en la capacité des usagers de se rétablir.	○ 1	○ 2	○ 3	○ 4	○ 5	○ 6	○ 7
13. … croient que les usagers ont la capacité de gérer leurs propres symptômes.	○ 1	○ 2	○ 3	○ 4	○ 5	○ 6	○ 7

37518

Les membres de l'équipe ...	Complètement en désaccord	Très en désaccord	Un peu en désaccord	Ni en désaccord ou en accord	Un peu en accord	Très en accord	Complètement en accord
14. … croient que les participants au programme peuvent effectuer leurs propres choix de vie en ce qui concerne où habiter, quand travailler, avec qui être amis, etc.	○ 1	○ 2	○ 3	○ 4	○ 5	○ 6	○ 7
15. … écoutent et respectent les décisions que prennent les participants à propos de leur traitement et de leurs soins.	○ 1	○ 2	○ 3	○ 4	○ 5	○ 6	○ 7
16. … demandent régulièrement aux usagers quels sont leurs intérêts et les choses qu'ils aimeraient faire dans la communauté.	○ 1	○ 2	○ 3	○ 4	○ 5	○ 6	○ 7
17. … encouragent les usagers à prendre des risques et à essayer de nouvelles choses.	○ 1	○ 2	○ 3	○ 4	○ 5	○ 6	○ 7
18. … offrent aux usagers des opportunités de discuter de leurs besoins et intérêts spirituels lorsqu'ils le souhaitent.	○ 1	○ 2	○ 3	○ 4	○ 5	○ 6	○ 7
19. … offrent aux usagers des opportunités de discuter de leurs besoins et intérêts sexuels lorsqu'ils le souhaitent.	○ 1	○ 2	○ 3	○ 4	○ 5	○ 6	○ 7
20. … aident les usagers à développer et planifier des objectifs de vie au-delà de la gestion des symptômes ou du seul fait d'atteindre une stabilité (ex. emploi, éducation, forme physique, être en relation avec la famille et les amis, passe-temps).	○ 1	○ 2	○ 3	○ 4	○ 5	○ 6	○ 7
21. …assistent systématiquement les usagers pour obtenir du travail.	○ 1	○ 2	○ 3	○ 4	○ 5	○ 6	○ 7
22. … aident activement les usagers pour qu'ils s'impliquent dans des activités non liées à la santé mentale, comme des groupes religieux, d'éducation aux adultes, des sports ou des passe-temps.	○ 1	○ 2	○ 3	○ 4	○ 5	○ 6	○ 7
23. … mettent tout en œuvre pour aider les usagers à inclure des personnes qui sont importantes pour eux dans la planification de leur traitement et de leur rétablissement (comme la famille, des amis, le clergé, ou un employeur).	○ 1	○ 2	○ 3	○ 4	○ 5	○ 6	○ 7
24. … présentent le plus souvent possible aux usagers des personnes en rétablissement qui peuvent leur servir de modèles ou de mentors en les référant à des groupes d'entraide et de soutien.	○ 1	○ 2	○ 3	○ 4	○ 5	○ 6	○ 7
25. … mettent le plus souvent possible les usagers en contact avec des programmes ou des groupes d'entraide, de soutien entre pairs ou de défense des droits en santé mentale.	○ 1	○ 2	○ 3	○ 4	○ 5	○ 6	○ 7
26. … aident concrètement les usagers à trouver des moyens de rendre service à leur communauté (ex. bénévolat, travaux communautaires, surveillance, corvée de nettoyage dans le quartier).	○ 1	○ 2	○ 3	○ 4	○ 5	○ 6	○ 7
27. … parlent avec les usagers à propos de ce que ça prendrait pour compléter ou quitter le programme.	○ 1	○ 2	○ 3	○ 4	○ 5	○ 6	○ 7
28. … aident les usagers à faire le suivi de leurs progrès en fonction de leurs objectifs personnels.	○ 1	○ 2	○ 3	○ 4	○ 5	○ 6	○ 7

Les membres de l'équipe ...	Complètement en désaccord	Très en désaccord	Un peu en désaccord	Ni en désaccord ou en accord	Un peu en accord	Très en accord	Complètement en accord
29. … ont comme rôle premier d'assister la personne à accomplir ses propres objectifs personnels et ses aspirations.	○ 1	○ 2	○ 3	○ 4	○ 5	○ 6	○ 7
30. … assistent régulièrement à des formations sur la diversité et les différences culturelles.	○ 1	○ 2	○ 3	○ 4	○ 5	○ 6	○ 7
31. … connaissent les activités des groupes d'entraide et communautaires de leur milieu.	○ 1	○ 2	○ 3	○ 4	○ 5	○ 6	○ 7
32. … sont diversifiés sur les plans de la culture, de l'ethnicité, du style de vie et des intérêts.	○ 1	○ 2	○ 3	○ 4	○ 5	○ 6	○ 7

g) Confiance

	Complètement en désaccord	Très en désaccord	Un peu en désaccord	Ni en désaccord ou en accord	Un peu en accord	Très en accord	Complètement en accord
1. Dans l'équipe, nous avons un respect profond et mutuel de la compétence de l'autre.	○ 1	○ 2	○ 3	○ 4	○ 5	○ 6	○ 7
2. Chaque membre de l'équipe fait montre d'intégrité absolue.	○ 1	○ 2	○ 3	○ 4	○ 5	○ 6	○ 7
3. Nous comptons sur la totale sincérité de chacun de nous.	○ 1	○ 2	○ 3	○ 4	○ 5	○ 6	○ 7
4. Nous sommes tous assurés de la confiance inébranlable que nous avons les uns envers les autres.	○ 1	○ 2	○ 3	○ 4	○ 5	○ 6	○ 7

h) Interdépendance d'équipe

	Complètement en désaccord	Très en désaccord	Un peu en désaccord	Ni en désaccord ou en accord	Un peu en accord	Très en accord	Complètement en accord
1. Mon travail est conçu de telle sorte que je dois interagir avec mes collègues de travail afin de travailler efficacement.	○ 1	○ 2	○ 3	○ 4	○ 5	○ 6	○ 7
2. La nature de mon travail m'oblige à travailler de concert avec mes collègues de travail pour effectuer des tâches spécifiques.	○ 1	○ 2	○ 3	○ 4	○ 5	○ 6	○ 7
3. J'ai souvent besoin de travailler directement avec mes collègues afin de bien faire mon travail.	○ 1	○ 2	○ 3	○ 4	○ 5	○ 6	○ 7
4. Si je ne m'implique pas dans les interactions avec mes collègues, il m'est difficile de bien faire mon travail.	○ 1	○ 2	○ 3	○ 4	○ 5	○ 6	○ 7
5. Mon travail m'oblige à coordonner mes actions avec celles de mes collègues.	○ 1	○ 2	○ 3	○ 4	○ 5	○ 6	○ 7
6. Je suis incapable de faire mon travail efficacement si certains collègues ne sont pas disponibles.	○ 1	○ 2	○ 3	○ 4	○ 5	○ 6	○ 7
7. Mes collègues et moi-même dépendons de nos actions réciproques pour faire notre travail.	○ 1	○ 2	○ 3	○ 4	○ 5	○ 6	○ 7
8. Mes collègues ne peuvent pas mener à bien leur travail s'ils ne reçoivent pas d'information de ma part.	○ 1	○ 2	○ 3	○ 4	○ 5	○ 6	○ 7
9. Mon travail nécessite que j'utilise les ressources fournies par des collègues afin d'exécuter mes tâches.	○ 1	○ 2	○ 3	○ 4	○ 5	○ 6	○ 7

37518

h) Interdépendance d'équipe (suite)

	Complètement en désaccord	Très en désaccord	Un peu en désaccord	Ni en désaccord ou en accord	Un peu en accord	Très en accord	Complètement en accord
10. Je compte sur mes collègues pour obtenir des informations afin que je puisse atteindre le niveau de performance souhaité au travail.	○ 1	○ 2	○ 3	○ 4	○ 5	○ 6	○ 7
11. Je compte sur mes collègues pour obtenir le matériel ou les outils dont j'ai besoin pour atteindre le niveau de performance souhaité au travail.	○ 1	○ 2	○ 3	○ 4	○ 5	○ 6	○ 7
12. Je dépends de la contribution de mes collègues pour compléter mon travail.	○ 1	○ 2	○ 3	○ 4	○ 5	○ 6	○ 7
13. Mon patron encourage ses employés à se concentrer sur les objectifs fixés au niveau de l'équipe plutôt que sur les objectifs individuels.	○ 1	○ 2	○ 3	○ 4	○ 5	○ 6	○ 7
14. Mon patron fixe des objectifs qui dépendent de la performance de plusieurs personnes.	○ 1	○ 2	○ 3	○ 4	○ 5	○ 6	○ 7
15. Il est important pour mon groupe de travail de se fixer des objectifs d'équipe.	○ 1	○ 2	○ 3	○ 4	○ 5	○ 6	○ 7
16. Mes collègues et moi-même avons des objectifs de travail identiques ou similaires.	○ 1	○ 2	○ 3	○ 4	○ 5	○ 6	○ 7
17. Mes collègues et moi avons des objectifs de travail communs.	○ 1	○ 2	○ 3	○ 4	○ 5	○ 6	○ 7
18. Les objectifs que je me fixe au travail dépendent de la progression de mes collègues.	○ 1	○ 2	○ 3	○ 4	○ 5	○ 6	○ 7

i) Réflexivité d'équipe

	Complètement en désaccord	Très en désaccord	Un peu en désaccord	Ni en désaccord ou en accord	Un peu en accord	Très en accord	Complètement en accord
1. L'équipe revoit souvent ses objectifs.	○ 1	○ 2	○ 3	○ 4	○ 5	○ 6	○ 7
2. Les méthodes utilisées par l'équipe pour mener à bien le travail sont souvent discutées.	○ 1	○ 2	○ 3	○ 4	○ 5	○ 6	○ 7
3. Nous discutons régulièrement de notre efficacité à travailler ensemble.	○ 1	○ 2	○ 3	○ 4	○ 5	○ 6	○ 7
4. Dans cette équipe, nous modifions nos objectifs à la lumière des circonstances changeantes.	○ 1	○ 2	○ 3	○ 4	○ 5	○ 6	○ 7

37518

j) Partage des connaissances

	Complètement en désaccord	Très en désaccord	Un peu en désaccord	Ni en désaccord ou en accord	Un peu en accord	Très en accord	Complètement en accord
1. Je partage mes rapports de travail et les documents officiels avec les autres membres de mon équipe.	○ 1	○ 2	○ 3	○ 4	○ 5	○ 6	○ 7
2. Je fournis mes manuels, mes méthodes et des exemples (ou modèles) aux membres de mon équipe.	○ 1	○ 2	○ 3	○ 4	○ 5	○ 6	○ 7
3. Je partage mon expérience ou mon savoir-faire avec les membres de mon équipe.	○ 1	○ 2	○ 3	○ 4	○ 5	○ 6	○ 7
4. Je fournis mes connaissances et mon expérience aux membres de l'équipe.	○ 1	○ 2	○ 3	○ 4	○ 5	○ 6	○ 7
5. Je partage l'expertise qui provient de ma formation avec les autres membres de l'équipe.	○ 1	○ 2	○ 3	○ 4	○ 5	○ 6	○ 7

k) Engagement affectif envers l'équipe

**Les membres de l'équipe ...**	Complètement en désaccord	Très en désaccord	Un peu en désaccord	Ni en désaccord ou en accord	Un peu en accord	Très en accord	Complètement en accord
1. ...se sentent émotionnellement attachés à l'équipe.	○ 1	○ 2	○ 3	○ 4	○ 5	○ 6	○ 7
2. ...ont un fort sentiment d'appartenance à l'équipe.	○ 1	○ 2	○ 3	○ 4	○ 5	○ 6	○ 7
3. …ressentent les problèmes de l'équipe comme étant les leurs.	○ 1	○ 2	○ 3	○ 4	○ 5	○ 6	○ 7
4. ...ont l'impression de faire partie d'une famille.	○ 1	○ 2	○ 3	○ 4	○ 5	○ 6	○ 7

l) Tâches et adaptation

	Complètement en désaccord	Très en désaccord	Un peu en désaccord	Ni en désaccord ou en accord	Un peu en accord	Très en accord	Complètement en accord
1. Je coordonne mon travail avec mes collègues.	○ 1	○ 2	○ 3	○ 4	○ 5	○ 6	○ 7
2. Je communique efficacement avec mes collègues.	○ 1	○ 2	○ 3	○ 4	○ 5	○ 6	○ 7
3. Je fournis de l'aide à mes collègues lorsque demandé ou nécessaire.	○ 1	○ 2	○ 3	○ 4	○ 5	○ 6	○ 7
4. Je gère efficacement les changements qui affectent mon équipe (ex. nouveaux membres).	○ 1	○ 2	○ 3	○ 4	○ 5	○ 6	○ 7
5. J'acquiers de nouvelles compétences ou j'endosse de nouveaux rôles pour faire face aux changements dans la façon dont mon équipe fonctionne.	○ 1	○ 2	○ 3	○ 4	○ 5	○ 6	○ 7
6. Je réponds de façon constructive aux changements dans la façon dont mon équipe travaille.	○ 1	○ 2	○ 3	○ 4	○ 5	○ 6	○ 7
7. Je suggère des moyens pour rendre mon équipe de travail plus efficace.	○ 1	○ 2	○ 3	○ 4	○ 5	○ 6	○ 7
8. Je développe des méthodes nouvelles et améliorées pour aider mon équipe de travail à mieux performer.	○ 1	○ 2	○ 3	○ 4	○ 5	○ 6	○ 7

l) Tâches et adaptation (suite)

	Complètement en désaccord	Très en désaccord	Un peu en désaccord	Ni en désaccord ou en accord	Un peu en accord	Très en accord	Complètement en accord
9. J'améliore la façon dont mon équipe de travail fait les choses.	○ 1	○ 2	○ 3	○ 4	○ 5	○ 6	○ 7
10. Je m'acquitte des parties essentielles de mon travail.	○ 1	○ 2	○ 3	○ 4	○ 5	○ 6	○ 7
11. Je complète correctement mes principales tâches en employant les procédures courantes.	○ 1	○ 2	○ 3	○ 4	○ 5	○ 6	○ 7
12. Je m'assure que mes tâches sont convenablement terminées.	○ 1	○ 2	○ 3	○ 4	○ 5	○ 6	○ 7
13. Je m'adapte aux changements apportés aux principales tâches.	○ 1	○ 2	○ 3	○ 4	○ 5	○ 6	○ 7
14. Je m'adapte aux changements dans la façon dont je dois accomplir mes principales tâches.	○ 1	○ 2	○ 3	○ 4	○ 5	○ 6	○ 7
15. J'acquiers de nouvelles compétences pour m'aider à m'adapter aux changements dans mes principales tâches.	○ 1	○ 2	○ 3	○ 4	○ 5	○ 6	○ 7
16. J'entreprends de modifier pour le mieux la manière d'effectuer mes principales tâches.	○ 1	○ 2	○ 3	○ 4	○ 5	○ 6	○ 7
17. Je trouve des idées pour améliorer ma façon d'accomplir mes tâches essentielles.	○ 1	○ 2	○ 3	○ 4	○ 5	○ 6	○ 7
18. J'effectue des modifications à la façon dont mes tâches essentielles sont faites.	○ 1	○ 2	○ 3	○ 4	○ 5	○ 6	○ 7

m) Identification multidimensionnelle

	Complètement en désaccord	Très en désaccord	Un peu en désaccord	Ni en désaccord ou en accord	Un peu en accord	Très en accord	Complètement en accord
1. Je perçois cette équipe comme étant une partie de moi-même.	○ 1	○ 2	○ 3	○ 4	○ 5	○ 6	○ 7
2. Je me vois comme étant tout à fait différent des autres membres de mon équipe.	○ 1	○ 2	○ 3	○ 4	○ 5	○ 6	○ 7
3. Lorsque quelqu'un critique mon équipe, je le ressens comme une insulte personnelle.	○ 1	○ 2	○ 3	○ 4	○ 5	○ 6	○ 7
4. Lorsque je parle de mon équipe, je dis généralement " nous " plutôt que " eux ".	○ 1	○ 2	○ 3	○ 4	○ 5	○ 6	○ 7
5. Je ressens les succès de mon équipe comme étant les miens.	○ 1	○ 2	○ 3	○ 4	○ 5	○ 6	○ 7
6. Je ressens les échecs de mon équipe comme étant les miens.	○ 1	○ 2	○ 3	○ 4	○ 5	○ 6	○ 7
7. Lorsque quelqu'un louange mon équipe, je le ressens comme un compliment personnel.	○ 1	○ 2	○ 3	○ 4	○ 5	○ 6	○ 7
8. J'apprécie le fait d'interagir avec les membres de cette équipe.	○ 1	○ 2	○ 3	○ 4	○ 5	○ 6	○ 7
9. Je n'aime pas la plupart des membres de cette équipe.	○ 1	○ 2	○ 3	○ 4	○ 5	○ 6	○ 7
10. Je ressens des liens étroits avec les membres de mon équipe.	○ 1	○ 2	○ 3	○ 4	○ 5	○ 6	○ 7
11. Je suis fier d'appartenir à mon équipe.	○ 1	○ 2	○ 3	○ 4	○ 5	○ 6	○ 7

m) Identification multidimensionnelle (suite)

	Complètement en désaccord	Très en désaccord	Un peu en désaccord	Ni en désaccord ou en accord	Un peu en accord	Très en accord	Complètement en accord
12. En général, je suis fier d'être un membre de mon équipe.	○ 1	○ 2	○ 3	○ 4	○ 5	○ 6	○ 7
13. Dans l'ensemble, j'ai l'impression que mon appartenance à cette équipe n'en vaut pas la peine.	○ 1	○ 2	○ 3	○ 4	○ 5	○ 6	○ 7
14. Je me sens bien par rapport à mon appartenance à cette équipe.	○ 1	○ 2	○ 3	○ 4	○ 5	○ 6	○ 7
15. Globalement, mon appartenance à cette équipe est bien vue par les autres.	○ 1	○ 2	○ 3	○ 4	○ 5	○ 6	○ 7
16. En général, les autres respectent mon appartenance à cette équipe.	○ 1	○ 2	○ 3	○ 4	○ 5	○ 6	○ 7
17. Je suis activement impliqué dans mon équipe.	○ 1	○ 2	○ 3	○ 4	○ 5	○ 6	○ 7
18. Dans l'ensemble, mon appartenance à cette équipe n'affecte pas comment je me sens par rapport à moi-même.	○ 1	○ 2	○ 3	○ 4	○ 5	○ 6	○ 7
19. Mon appartenance à cette équipe est une importante part de la personne que je suis.	○ 1	○ 2	○ 3	○ 4	○ 5	○ 6	○ 7
20. Je pense souvent au fait que je suis membre de cette équipe.	○ 1	○ 2	○ 3	○ 4	○ 5	○ 6	○ 7
21. Le fait que je sois membre de cette équipe est une partie importante de mon identité.	○ 1	○ 2	○ 3	○ 4	○ 5	○ 6	○ 7
22. Être membre de cette équipe est une partie importante de la façon dont je me perçois.	○ 1	○ 2	○ 3	○ 4	○ 5	○ 6	○ 7
23. Je perçois cette profession ou discipline comme étant une partie de moi-même.	○ 1	○ 2	○ 3	○ 4	○ 5	○ 6	○ 7
24. Je me vois comme étant tout à fait différent des autres membres de ma profession ou discipline.	○ 1	○ 2	○ 3	○ 4	○ 5	○ 6	○ 7
25. Lorsque quelqu'un critique ma profession ou discipline, je le ressens comme une insulte personnelle.	○ 1	○ 2	○ 3	○ 4	○ 5	○ 6	○ 7
26. Lorsque je parle de ma profession ou discipline, je dis généralement " nous " plutôt que " eux ".	○ 1	○ 2	○ 3	○ 4	○ 5	○ 6	○ 7
27. Je ressens les succès de ma profession ou discipline comme étant les miens.	○ 1	○ 2	○ 3	○ 4	○ 5	○ 6	○ 7
28. Je ressens les échecs de ma profession ou discipline comme étant les miens.	○ 1	○ 2	○ 3	○ 4	○ 5	○ 6	○ 7
29. Lorsque quelqu'un louange ma profession ou discipline, je le ressens comme un compliment personnel.	○ 1	○ 2	○ 3	○ 4	○ 5	○ 6	○ 7
30. J'apprécie le fait d'interagir avec les membres de cette profession ou discipline.	○ 1	○ 2	○ 3	○ 4	○ 5	○ 6	○ 7
31. Je n'aime pas la plupart des membres de cette profession ou discipline.	○ 1	○ 2	○ 3	○ 4	○ 5	○ 6	○ 7
32. Je ressens des liens étroits avec les autres membres de ma profession ou discipline.	○ 1	○ 2	○ 3	○ 4	○ 5	○ 6	○ 7
33. Je suis fier d'appartenir à ma profession ou discipline.	○ 1	○ 2	○ 3	○ 4	○ 5	○ 6	○ 7

m) Identification multidimensionnelle (suite)

	Complètement en désaccord	Très en désaccord	Un peu en désaccord	Ni en désaccord ou en accord	Un peu en accord	Très en accord	Complètement en accord
34. En général, je suis fier d'être un membre de ma profession ou discipline.	○ 1	○ 2	○ 3	○ 4	○ 5	○ 6	○ 7
35. Dans l'ensemble, j'ai l'impression que mon appartenance à cette profession ou discipline n'en vaut pas la peine.	○ 1	○ 2	○ 3	○ 4	○ 5	○ 6	○ 7
36. Je me sens bien par rapport à mon appartenance à cette profession ou discipline.	○ 1	○ 2	○ 3	○ 4	○ 5	○ 6	○ 7
37. Globalement, mon appartenance à cette profession ou discipline est bien vue par les autres.	○ 1	○ 2	○ 3	○ 4	○ 5	○ 6	○ 7
38. En général, les autres respectent mon appartenance à cette profession ou discipline.	○ 1	○ 2	○ 3	○ 4	○ 5	○ 6	○ 7
39. Je suis activement impliqué dans ma profession ou discipline.	○ 1	○ 2	○ 3	○ 4	○ 5	○ 6	○ 7
40. Dans l'ensemble, mon appartenance à cette profession ou discipline n'affecte pas la façon dont je me sens par rapport à moi-même.	○ 1	○ 2	○ 3	○ 4	○ 5	○ 6	○ 7
41. Mon appartenance à cette profession ou discipline est une part importante de ce que je suis.	○ 1	○ 2	○ 3	○ 4	○ 5	○ 6	○ 7
42. Je pense souvent au fait que je suis membre de cette profession ou discipline.	○ 1	○ 2	○ 3	○ 4	○ 5	○ 6	○ 7
43. Le fait que je sois membre de cette profession ou discipline est une partie importante de mon identité.	○ 1	○ 2	○ 3	○ 4	○ 5	○ 6	○ 7
44. Être membre de cette profession ou discipline est une partie importante de la façon dont je me perçois.	○ 1	○ 2	○ 3	○ 4	○ 5	○ 6	○ 7

n) Identification multifocale

	Complètement en désaccord	Très en désaccord	Un peu en désaccord	Ni en désaccord ou en accord	Un peu en accord	Très en accord	Complètement en accord
1. Je m'identifie comme un membre de mon équipe.	○ 1	○ 2	○ 3	○ 4	○ 5	○ 6	○ 7
2. Être membre de mon équipe reflète bien ma personnalité.	○ 1	○ 2	○ 3	○ 4	○ 5	○ 6	○ 7
3. J'aime travailler pour mon équipe.	○ 1	○ 2	○ 3	○ 4	○ 5	○ 6	○ 7
4. J'ai des réticences lorsque je pense à mon équipe.	○ 1	○ 2	○ 3	○ 4	○ 5	○ 6	○ 7
5. Mon équipe est bien vue par les autres.	○ 1	○ 2	○ 3	○ 4	○ 5	○ 6	○ 7
6. Je fais plus pour mon équipe que ce qui est absolument nécessaire.	○ 1	○ 2	○ 3	○ 4	○ 5	○ 6	○ 7
7. Je m'identifie comme un membre de ma profession ou discipline.	○ 1	○ 2	○ 3	○ 4	○ 5	○ 6	○ 7

n) Identification multifocale (suite)

	Complètement en désaccord	Très en désaccord	Un peu en désaccord	Ni en désaccord ou en accord	Un peu en accord	Très en accord	Complètement en accord
8. Être membre de ma profession ou discipline reflète bien ma personnalité.	○ 1	○ 2	○ 3	○ 4	○ 5	○ 6	○ 7
9. J'aime travailler pour ma profession ou discipline.	○ 1	○ 2	○ 3	○ 4	○ 5	○ 6	○ 7
10. J'ai des réticences lorsque je pense à ma profession ou discipline.	○ 1	○ 2	○ 3	○ 4	○ 5	○ 6	○ 7
11. Ma profession ou discipline est bien vue par les autres.	○ 1	○ 2	○ 3	○ 4	○ 5	○ 6	○ 7
12. Je fais plus pour ma profession ou discipline que ce qui est absolument nécessaire.	○ 1	○ 2	○ 3	○ 4	○ 5	○ 6	○ 7

o) Climat

	Complètement en désaccord	Très en désaccord	Un peu en désaccord	Ni en désaccord ou en accord	Un peu en accord	Très en accord	Complètement en accord
1. Notre attitude est que " nous sommes tous dans le même bateau ".	○ 1	○ 2	○ 3	○ 4	○ 5	○ 6	○ 7
2. Les gens s'informent les uns les autres des questions relatives au travail dans l'équipe.	○ 1	○ 2	○ 3	○ 4	○ 5	○ 6	○ 7
3. Les gens se sentent compris et acceptés par les autres.	○ 1	○ 2	○ 3	○ 4	○ 5	○ 6	○ 7
4. Des efforts réels sont faits pour partager l'information dans toute l'équipe.	○ 1	○ 2	○ 3	○ 4	○ 5	○ 6	○ 7
5. Il y a beaucoup de concessions mutuelles.	○ 1	○ 2	○ 3	○ 4	○ 5	○ 6	○ 7
6. Nous restons en contact les uns avec les autres au sein de l'équipe.	○ 1	○ 2	○ 3	○ 4	○ 5	○ 6	○ 7
7. L'équipe est toujours en train de développer des nouvelles solutions aux problèmes qui surviennent.	○ 1	○ 2	○ 3	○ 4	○ 5	○ 6	○ 7
8. L'équipe est ouverte et sait réagir au changement.	○ 1	○ 2	○ 3	○ 4	○ 5	○ 6	○ 7
9. Les gens de l'équipe sont toujours en train de chercher de nouvelles façons d'envisager les problèmes.	○ 1	○ 2	○ 3	○ 4	○ 5	○ 6	○ 7
10. Les membres de l'équipe offrent et partagent des ressources pour aider à appliquer des idées nouvelles.	○ 1	○ 2	○ 3	○ 4	○ 5	○ 6	○ 7
11. Les membres de l'équipe offrent un soutien pratique aux idées nouvelles et à leur application.	○ 1	○ 2	○ 3	○ 4	○ 5	○ 6	○ 7
12. Les membres de votre équipe offrent des idées utiles et une aide pratique pour vous permettre de faire le travail du mieux que vous le pouvez.	○ 1	○ 2	○ 3	○ 4	○ 5	○ 6	○ 7
13. Les membres de l'équipe sont prêts à remettre en question les fondements de l'activité de l'équipe.	○ 1	○ 2	○ 3	○ 4	○ 5	○ 6	○ 7
14. L'équipe évalue de façon critique les faiblesses potentielles de ce qu'elle fait afin d'obtenir les meilleurs résultats possibles.	○ 1	○ 2	○ 3	○ 4	○ 5	○ 6	○ 7

37518

o) Climat (suite)

	Complètement en désaccord	Très en désaccord	Un peu en désaccord	Ni en désaccord ou en accord	Un peu en accord	Très en accord	Complètement en accord
15. Les membres de l'équipe s'inspirent des idées des uns et des autres afin d'obtenir les meilleurs résultats possibles.	○ 1	○ 2	○ 3	○ 4	○ 5	○ 6	○ 7

	Pas du tout	Très peu	Un peu	Ni peu, Ni suffisamment	Suffisamment	Beaucoup	Totalement
16. Jusqu'à quel point les objectifs de votre équipe sont-ils clairs pour vous?	○ 1	○ 2	○ 3	○ 4	○ 5	○ 6	○ 7
17. Jusqu'à quel point êtes-vous d'accord avec ces objectifs?	○ 1	○ 2	○ 3	○ 4	○ 5	○ 6	○ 7
18. Jusqu'à quel point croyez-vous que les autres membres de l'équipe sont d'accord avec ces objectifs?	○ 1	○ 2	○ 3	○ 4	○ 5	○ 6	○ 7
19. Jusqu'à quel point croyez-vous que les membres de votre équipe sont engagés à atteindre ces objectifs?	○ 1	○ 2	○ 3	○ 4	○ 5	○ 6	○ 7

p) Rôle informationnel

Les équipes composées de personnes provenant de plusieurs professions ou disciplines sont très importantes. Nous avons tous une formation particulière et un domaine de connaissances qui ont le potentiel de contribuer au travail d'équipe. La prochaine liste d'énoncés décrit des activités qui réfèrent à votre expertise particulière. Évaluez votre degré de confiance quant à votre capacité à effectuer ces activités en associant à chaque activité n'importe quel chiffre entre 0% et 100% selon l'échelle suivante :

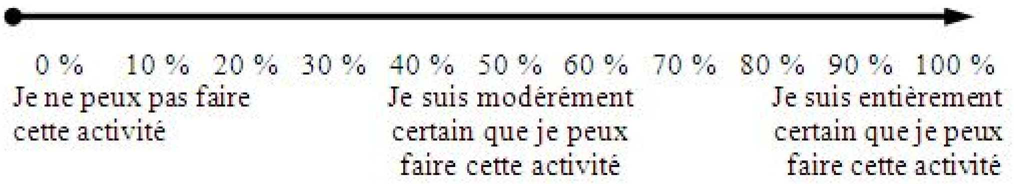

1. Prendre appui sur mon domaine d'expertise pour enrichir les discussions de l'équipe	☐☐☐	%
2. Bonifier le travail d'équipe par des interventions qui visent à mettre à profit mon expertise professionnelle	☐☐☐	%
3. Clarifier la nature de mon expertise professionnelle auprès des membres de mon équipe	☐☐☐	%
4. Conseiller les autres membres de l'équipe en intégrant les particularités de mon domaine d'expertise	☐☐☐	%
5. Montrer la contribution de mon domaine d'expertise lorsque l'équipe doit résoudre un problème	☐☐☐	%

37518

q) Conflits dans l'équipe

**À quelle fréquence...**	Jamais	Rarement	À l'occasion	Assez souvent	Souvent	Très souvent	Constamment
1. …votre équipe vit-elle des tensions concernant les relations interpersonnelles ?	○ 1	○ 2	○ 3	○ 4	○ 5	○ 6	○ 7
2. …y a-t-il des manifestations de colère dans votre équipe ?	○ 1	○ 2	○ 3	○ 4	○ 5	○ 6	○ 7
3. …votre équipe vit-elle des conflits mettant en jeu des émotions ?	○ 1	○ 2	○ 3	○ 4	○ 5	○ 6	○ 7
4. …votre équipe vit-elle des conflits sur le plan des idées ?	○ 1	○ 2	○ 3	○ 4	○ 5	○ 6	○ 7
5. …votre équipe vit-elle des divergences d'opinion concernant le travail ?	○ 1	○ 2	○ 3	○ 4	○ 5	○ 6	○ 7
6. …votre équipe vit-elle des opinions conflictuelles au sujet du travail à faire ?	○ 1	○ 2	○ 3	○ 4	○ 5	○ 6	○ 7
7. …votre équipe vit-elle des divergences d'opinion concernant "qui" fait "quoi" ?	○ 1	○ 2	○ 3	○ 4	○ 5	○ 6	○ 7
8. …votre équipe vit-elle des conflits concernant la nature des responsabilités associées aux tâches ?	○ 1	○ 2	○ 3	○ 4	○ 5	○ 6	○ 7
9. …votre équipe vit-elle des divergences d'opinion concernant l'allocation des ressources, par exemple le budget, le personnel, les outils de travail ?	○ 1	○ 2	○ 3	○ 4	○ 5	○ 6	○ 7

r) Production de connaissances

**Notre équipe...**	Jamais	Rarement	À l'occasion	Assez souvent	Souvent	Très souvent	Constamment
1. ... résout les problèmes de façon novatrice.	○ 1	○ 2	○ 3	○ 4	○ 5	○ 6	○ 7
2. ... produit des concepts originaux pour les services que nous fournissons.	○ 1	○ 2	○ 3	○ 4	○ 5	○ 6	○ 7
3. ... génère des idées uniques qui définissent notre travail et le rend unique.	○ 1	○ 2	○ 3	○ 4	○ 5	○ 6	○ 7
4. ... crée des solutions innovantes pour faire face à la complexité de notre travail.	○ 1	○ 2	○ 3	○ 4	○ 5	○ 6	○ 7
5. ... conçoit de nouvelles stratégies pour faire le travail.	○ 1	○ 2	○ 3	○ 4	○ 5	○ 6	○ 7

37518

s) L'intégration des connaissances

Notre équipe...	Jamais	Rarement	À l'occasion	Assez souvent	Souvent	Très souvent	Constamment
1. ... assimile des connaissances à l'extérieur à l'équipe afin que nous puissions prendre de meilleures décisions.	○ 1	○ 2	○ 3	○ 4	○ 5	○ 6	○ 7
2. ... intègre des éléments de rapports passés, et qui résument les apprentissages.	○ 1	○ 2	○ 3	○ 4	○ 5	○ 6	○ 7
3. ... utilise les informations qui expliquent pourquoi les expériences du passé ont échoué ou rencontré des difficultés.	○ 1	○ 2	○ 3	○ 4	○ 5	○ 6	○ 7
4. ... intègre les connaissances formelles de ce qui a fonctionné par le passé.	○ 1	○ 2	○ 3	○ 4	○ 5	○ 6	○ 7
5. ... s'appuie sur des données de qualité pour planifier et exécuter le travail.	○ 1	○ 2	○ 3	○ 4	○ 5	○ 6	○ 7
6. ... intègre des informations provenant de diverses sources afin de maximiser la qualité de nos services.	○ 1	○ 2	○ 3	○ 4	○ 5	○ 6	○ 7
7. ... s'appuie sur des informations riches et variées afin de minimiser les risques.	○ 1	○ 2	○ 3	○ 4	○ 5	○ 6	○ 7
8. ... tire profit d'experts pour synthétiser les connaissances.	○ 1	○ 2	○ 3	○ 4	○ 5	○ 6	○ 7
9. ... interprète les informations complexes nécessaires à la réalisation du travail.	○ 1	○ 2	○ 3	○ 4	○ 5	○ 6	○ 7

t) Travail collaboratif

Dans mon équipe...	Jamais	Rarement	À l'occasion	Assez souvent	Souvent	Très souvent	Constamment
1. … nous nous donnons de l'information utile qui fait progresser le travail.	○ 1	○ 2	○ 3	○ 4	○ 5	○ 6	○ 7
2. … nous nous partageons des connaissances qui font avancer le travail.	○ 1	○ 2	○ 3	○ 4	○ 5	○ 6	○ 7
3. … nous nous comprenons lorsque nous parlons du travail à faire.	○ 1	○ 2	○ 3	○ 4	○ 5	○ 6	○ 7
4. … nous partageons des ressources qui aident à la réalisation des tâches.	○ 1	○ 2	○ 3	○ 4	○ 5	○ 6	○ 7
5. … nous nous communiquons nos idées au sujet du travail à faire.	○ 1	○ 2	○ 3	○ 4	○ 5	○ 6	○ 7
6. … nous faisons le travail que nous devons faire au bon moment.	○ 1	○ 2	○ 3	○ 4	○ 5	○ 6	○ 7
7. … nous faisons en sorte que nos tâches soient terminées à temps.	○ 1	○ 2	○ 3	○ 4	○ 5	○ 6	○ 7

37518

**Dans mon équipe…**	Jamais	Rarement	À l'occasion	Assez souvent	Souvent	Très souvent	Constamment
8. … nous nous ajustons afin de respecter les échéances.	○ 1	○ 2	○ 3	○ 4	○ 5	○ 6	○ 7
9. … nous faisons le point au sujet de la progression du travail.	○ 1	○ 2	○ 3	○ 4	○ 5	○ 6	○ 7
10. … nous nous échangeons de l'information au sujet de " qui fait quoi ".	○ 1	○ 2	○ 3	○ 4	○ 5	○ 6	○ 7
11. … nous discutons de l'échéancier.	○ 1	○ 2	○ 3	○ 4	○ 5	○ 6	○ 7
12. … nous anticipons les besoins des autres sans qu'ils aient à les exprimer.	○ 1	○ 2	○ 3	○ 4	○ 5	○ 6	○ 7
13. … nous réorganisons nos tâches instinctivement lorsque des changements sont nécessaires.	○ 1	○ 2	○ 3	○ 4	○ 5	○ 6	○ 7
14. … nous avons une compréhension implicite des tâches à effectuer.	○ 1	○ 2	○ 3	○ 4	○ 5	○ 6	○ 7

u) Soutien

	Complètement en désaccord	Très en désaccord	Un peu en désaccord	Ni en désaccord ou en accord	Un peu en accord	Très en accord	Complètement en accord
1. Généralement, je peux obtenir des ressources supplémentaires pour réaliser mon travail lorsque j'en ai besoin.	○ 1	○ 2	○ 3	○ 4	○ 5	○ 6	○ 7
2. J'ai accès aux ressources dont j'ai besoin pour bien faire mon travail.	○ 1	○ 2	○ 3	○ 4	○ 5	○ 6	○ 7
3. Je peux obtenir les ressources nécessaires pour appuyer de nouvelles idées.	○ 1	○ 2	○ 3	○ 4	○ 5	○ 6	○ 7
4. Je reçois l'aide et la supervision clinique dont j'ai besoin de la part de mon patron.	○ 1	○ 2	○ 3	○ 4	○ 5	○ 6	○ 7

3. Interdépendance Inter-équipes

Veuillez indiquer la fréquence à laquelle vous faites des plans d'intervention…

	Jamais	Rarement	À l'occasion	Assez souvent	Souvent	Très souvent	Constam-ment	**Ne s'applique pas**
… avec d'autres intervenants de votre propre organisation	1	2	3	4	5	6	7	N/A
… avec des intervenants provenant d'autres organisations	1	2	3	4	5	6	7	N/A

Veuillez indiquer la fréquence à laquelle vous êtes en interaction significative avec les équipes, organisations ou professionnels suivants.

	Jamais	Rarement	À l'occasion	Assez souvent	Souvent	Très souvent	Constam-ment	**Ne s'applique pas**
A) Omnipraticiens	1	2	3	4	5	6	7	N/A
B) Pharmaciens	1	2	3	4	5	6	7	N/A
C) Organismes communautaires en santé mentale								
- Centre de crise / suicide	1	2	3	4	5	6	7	N/A
- Soutien d'intensité variable (SIV)	1	2	3	4	5	6	7	N/A
- Groupe d'entraide (incluant centre de jour)	1	2	3	4	5	6	7	N/A
- Organisme communautaire d'hébergement (OCH)	1	2	3	4	5	6	7	N/A
D) Organismes intervenant en alcoolisme et toxicomanie	1	2	3	4	5	6	7	N/A
E) Autres organismes de la communauté (ex. aide matérielle)	1	2	3	4	5	6	7	N/A
F) Équipes de 1re ligne en santé mentale au CLSC								
- Équipe SM (équipe de base ou de suivi)	1	2	3	4	5	6	7	N/A
- Guichet d'accès en SM	1	2	3	4	5	6	7	N/A
- Équipe SIV	1	2	3	4	5	6	7	N/A
- Équipe des ressources non-institutionnelles (RNI) et RTF	1	2	3	4	5	6	7	N/A
G) Autres équipes en CLSC	1	2	3	4	5	6	7	N/A

37518

Veuillez indiquer la fréquence avec laquelle vous êtes en interaction significative avec les équipes, organisations ou professionnels suivants.

H) Équipes de santé mentale et autres équipes en CHSGS	Jamais	Rarement	À l'occasion	Assez souvent	Souvent	Très souvent	Constam-ment	**Ne s'applique pas**
- Urgences en SM	○ 1	○ 2	○ 3	○ 4	○ 5	○ 6	○ 7	○ N/A
- Unités d'hospitalisation en SM	○ 1	○ 2	○ 3	○ 4	○ 5	○ 6	○ 7	○ N/A
- Hôpital de jour en SM	○ 1	○ 2	○ 3	○ 4	○ 5	○ 6	○ 7	○ N/A
- Équipe de suivi intensif (SI) en SM	○ 1	○ 2	○ 3	○ 4	○ 5	○ 6	○ 7	○ N/A
- Cliniques externes	○ 1	○ 2	○ 3	○ 4	○ 5	○ 6	○ 7	○ N/A
- Professionnels et équipes autres qu'en psychiatrie - santé physique	○ 1	○ 2	○ 3	○ 4	○ 5	○ 6	○ 7	○ N/A
I) Équipes et professionnels en CH psychiatrique								
- Urgence	○ 1	○ 2	○ 3	○ 4	○ 5	○ 6	○ 7	○ N/A
- MEL (ou service de triage et de consultation clinique)	○ 1	○ 2	○ 3	○ 4	○ 5	○ 6	○ 7	○ N/A
- Service d'intervention rapide (quelques séances en externe pour éviter l'hospitalisation)	○ 1	○ 2	○ 3	○ 4	○ 5	○ 6	○ 7	○ N/A
- Psychiatres répondants	○ 1	○ 2	○ 3	○ 4	○ 5	○ 6	○ 7	○ N/A
- Unités d'hospitalisation	○ 1	○ 2	○ 3	○ 4	○ 5	○ 6	○ 7	○ N/A
- Hôpital de jour	○ 1	○ 2	○ 3	○ 4	○ 5	○ 6	○ 7	○ N/A
- Cliniques externes (ex. troubles de la personnalité, de l'humeur)	○ 1	○ 2	○ 3	○ 4	○ 5	○ 6	○ 7	○ N/A
- Programme / service pour premier épisode de psychose	○ 1	○ 2	○ 3	○ 4	○ 5	○ 6	○ 7	○ N/A
- Réadaptation sociale et spécialisée (ex. emploi, activités de réadaptation)	○ 1	○ 2	○ 3	○ 4	○ 5	○ 6	○ 7	○ N/A
- Hébergement spécialisé - ressources non institutionnelles (RNI) et ressources de type familial (RTF)	○ 1	○ 2	○ 3	○ 4	○ 5	○ 6	○ 7	○ N/A
- Suivi intensif (SI)	○ 1	○ 2	○ 3	○ 4	○ 5	○ 6	○ 7	○ N/A
J) Équipes et intervenants d'autres secteurs (ex. emploi, éducation)	○ 1	○ 2	○ 3	○ 4	○ 5	○ 6	○ 7	○ N/A

Nous vous remercions de votre collaboration à ce projet de recherche !
