# Supplementary material for: Variables associated with job satisfaction among mental health professionals
Source: PLoS One. 2018 Oct 18;13(10):e0205963. doi: 10.1371/journal.pone.0205963 (PMC6193708; doi:10.1371/journal.pone.0205963)
Supplement: S2 File — (DOCX) [file pone.0205963.s002.docx]

**Supplementary file 2**

**Managers Questionnaire**

**Primary care teams (sections/mains topics)**

1. **Individual characteristics**
2. **Age**
3. **Gender**
4. **Position**
5. **Seniority**
6. **Number of professional in the team**
7. **Client characteristics**
8. **Age**
9. **Mother tongue**
10. **Income**
11. **Diagnosis**
12. **Other clinical variables (suicidal ideation, problems with justice system, high services users, etc.)**
13. **Utilization of services during the last 12 months (general practitioner, psychologists, emergency, etc.)**
14. **% clients referred to other services (specialized services, mental health community organizations, etc.)**
15. **Team characteristics**
16. **Type of teams**
17. **Number of professionals in the team (psychiatrists, general practitioners, nurses, psychologists, social workers, psycho-educators, occupational therapists, substance use disorder specialists)**
18. **Clinical Activities**
19. **% time allocated per week to evaluation, treatment or intervention**
20. **% of time at the office; in other place (at home; in the community, in other organisations)**
21. **Case load per professional**
22. **Frequency of client follow-up**
23. **Duration of client follow-up**
24. **Frequency of clinical approaches used (stepped care, cognitive behavioral approach, motivational interviewing, strengths model, care pathways, recovery approach, self-management)**
25. **Frequency of clinical evaluation tools used (MH disorder screening tools, MH disorder assessment tools, substance use screening tools, substance use assessment tools, assesment tools for patient satisfaction, clinical feedback procedures, clinical protocols or best practice guidelines, intra-organizational referral procedures, and shared clinical records)**
26. **Work role Performance**
27. **Organizational culture**
28. **Level of implementation re network integration strategies (taff supervision level, systematic patient monitoring, waiting list management procedures, liaison officers, shared staff, service availability for substance use disorders, strategic planning, service agreements, joint training, inter-organizational referral procedures, and network resource directories)**
29. **Frequency and satisfaction of interactions involving network teams or organizations**

- **with other HSSC teams (one-stop services, general services, intensive case management, other)**
- **with specialized services (respondent-psychiatrists, emergency rooms, day hospitals, hospital units, assertive community treatment, out-patient clinics, other)**
- **with other network organizations (general practitioners in medical clinics, pharmacists, crisis centers, day centers, non-mental health community organizations, substance use disorder rehabilitation centers, other)**

1. **Assessment of mental health services in the network**
2. **Accessibility (day, evening and week end)**
3. **General practitioners in medical clinics**
4. **Substance use disorders**
5. **Wait time in the emergency room**
6. **Alternatives to hospitalization for clients with severe mental disorders**
7. **Services for clients with common mental health disorders**
8. **Community-based organizations**
9. **Mental health specialized services**

**Managers Questionnaires**

**Specialized services teams (sections/mains topics)**

1. **Individual characteristics**
2. **Age**
3. **Gender**
4. **Position**
5. **Seniority**
6. **Number of professional in the team**
7. **Client characteristics**
8. **Age**
9. **Mother tongue**
10. **Income**
11. **Diagnosis**
12. **Other clinical variables (suicidal ideation, problems with justice system, high services users, etc.)**
13. **Utilization of services during the last 12 months (general practitioner, psychologists, emergency, etc.)**
14. **% clients referred to other services (specialized services, mental health community organizations, etc.)**
15. **Team characteristics**
16. **Type of teams**
17. **Number of professionals in the team (psychiatrists, general practitioners, nurses, psychologists, social workers, psycho-educators, occupational therapists, substance use disorder specialists)**
18. **Clinical Activities**
19. **% time allocated per week to evaluation, treatment or intervention**
20. **Case load per professional**
21. **Frequency of client follow-up**
22. **Duration of client follow-up**
23. **Frequency of clinical approaches used (stepped care, cognitive behavioral approach, motivational interviewing, strengths model, care pathways, recovery approach, self-management)**
24. **Frequency of clinical evaluation tools used (MH disorder screening tools, MH disorder assessment tools, substance use screening tools, substance use assessment tools, assesment tools for patient satisfaction, clinical feedback procedures, clinical protocols or best practice guidelines, intra-organizational referral procedures, and shared clinical records)**
25. **Work role Performance**
26. **Organizational culture**
27. **Level of implementation re network integration strategies (taff supervision level, systematic patient monitoring, waiting list management procedures, liaison officers, shared staff, service availability for substance use disorders, strategic planning, service agreements, joint training, inter-organizational referral procedures, and network resource directories)**
28. **Frequency and satisfaction of interactions involving network teams or organizations**

- **with other specialized services (respondent-psychiatrists, emergency rooms, day hospitals, hospital units, assertive community treatment, out-patient clinics, other)**
- **with primary care teams (one-stop services, general services, intensive case management, other)**
- **with other network organizations (general practitioners in medical clinics, pharmacists, crisis centers, day centers, non-mental health community organizations, substance use disorder rehabilitation centers, other)**

1. **Assessment of mental health services in the network**
2. **Accessibility (day, evening and week end)**
3. **General practitioners in medical clinics**
4. **Substance use disorders**
5. **Wait time in the emergency room**
6. **Alternatives to hospitalization for clients with severe mental disorders**
7. **Services for clients with common mental health disorders**
8. **Community-based organizations**
9. **Mental health specialized services**
